# Immunological hotspots analyzed by docking simulations: evidence for a general mechanism in pemphigus vulgaris pathology and transformation

**DOI:** 10.1186/1471-2172-9-30

**Published:** 2008-06-19

**Authors:** Joo Chuan Tong, Animesh A Sinha

**Affiliations:** 1 Data Mining Department, Institute for Infocomm Research, 21 Heng Mui Keng Terrace, 119613, Singapore; 2 Center for Investigative Dermatology, Division of Dermatology and Cutaneous Sciences, College of Human Medicine, Michigan State University, 4120 Biomedical and Physical Sciences Building, East Lansing, MI 48824, USA

## Abstract

**Background:**

Pemphigus vulgaris (PV) is an acquired autoimmune blistering disorder in which greater than 80% of active patients produce autoantibodies to the desmosomal protein desmogelin 3 (Dsg3). As the disease progresses, 40–50% of patients may also develop reactivity to a second component of the desmosomal complex, desmogelin 1 (Dsg1). T cells are clearly required for the production of autoantibodies in PV. However, few T-cell specificities within Dsg3 or Dsg1 have been reported to date, and the precise role of T-cells in disease pathogenesis and evolution remains poorly understood. In particular, no studies have addressed the immunological mechanisms that underlie the observed clinical heterogeneity in pemphigus. We report here a structure-based technique for the screening of DRB1*0402-specific immunological (T-cell epitope) hotspots in both Dsg3 and Dsg1 glycoproteins.

**Results:**

High predictivity was obtained for DRB1*0402 (*r*^2 ^= 0.90, *s *= 1.20 kJ/mol, *q*^2 ^= 0.82, *s*_*press *_= 1.61 kJ/mol) predictive model, compared to experimental data. *In silico *mapping of the T-cell epitope repertoires in Dsg3 and Dsg1 glycoproteins revealed that the potential immunological hotspots of both target autoantigens are highly conserved, despite limited sequence identity (54% identical, 72% similar). A similar number of well-conserved (18%) high-affinity binders were predicted to exist within both Dsg3 and Dsg1, with analogous distribution of binding registers.

**Conclusion:**

This study provides interesting new insights into the possible mechanism for PV disease progression. Our data suggests that the potential T-cell epitope repertoires encoded in Dsg1 and Dsg3 is substantially overlapping, and it may be possible to apply a common, antigen-specific therapeutic strategy with efficacy across distinct clinical phases of disease.

## Background

Pemphigus vulgaris (PV) is characterized by the loss of normal epithelial cell-to-cell adhesion leading to blistering which may involve the mucous membranes, non-mucosal cutaneous surfaces, or both [1]. Pemphigus autoantibodies (autoAb) are mainly directed against the desmosomal glycoproteins desmoglein 3 (Dsg3) and desmoglein 1 (Dsg1), members of the cadherin superfamily of cell adhesion molecules [2].

Clinical evolution of disease expression is common in PV [3,4]. In early disease, a majority of PV patients develop autoantibodies to Dsg3 coincident with mucosal blisters. In later stages, significant proportions of patients develop additional lesions on non-mucosal cutaneous sites and exhibit non-cross-reactive immunity to both Dsg3 and Dsg1 [5].

Two immunologic phenomenon termed "antigen mimicry" [5] and "epitope spreading" [5-8] have been proposed as possible pathogenic mechanisms responsible for the shift in autoreactive lymphocyte (T- or B-cell) profile from Dsg3^+^/Dsg1^- ^to Dsg3^+^/Dsg1^+^. Antigen mimicry can be defined as the generation of lymphocyte (T- or B-cell) reactivity towards a protein due to its close structural similarity to unique exogenous antigens, or new determinants that have been generated endogenously [5]. Epitope spreading in the context of autoimmunity refers to the development of epitope-specific immune responses that are distinct from and non-cross-reactive with disease-inducing epitopes on the same (or different) protein secondary to the release of such a self-protein during an autoimmune response [8-10].

A close relationship between antigen mimicry and epitope spreading exists, with epitope spreading usually occurring after an initial episode of antigen mimicry [5]. Exogenous and endogenous antigens that may trigger cross-reactivity with self-proteins have not yet been defined in pemphigus [5]. While the modulation of autoantibody reactivities in the transformation of one disease subform into another has been actively explored [3-7], the role of T-cells underlying the evolution of autoreactive processes and epitope spreading remains poorly understood. To date, limited studies on T-cell specificities within PV have been reported [11-20]. The reported HLA associations with disease may serve to provide the genetic link that drives the evolving autoimmune responses in pemphigus. PV is known to be strongly associated with the HLA-DR allele DRB1*0402 [21-26]; it is present in more than 90% of Ashkenazi patients [27]. The DRB1*0402 allele is also common in other ethic backgrounds, including patients from France [28], Italy [29], Spain [30], Argentina [31] and Iran [32].

We have previously investigated the docking potentials of Dsg3 peptides to DRB1*0402 using a hybrid approach that integrates the strength of Monte Carlo simulations and homology modeling [33-37]. Consistent with experimental evidence [11], computational simulations reveal that a potentially large number of T-cell epitopes may be relevant in the pathogenesis of PV [33]. In the current study, we have extended our analysis to the Dsg1 glycoprotein and applied a new scoring scheme for identification of immunological (T-cell epitope) hotspots within both Dsg3 and Dsg1 self-antigens. *In silico *mapping of the T-cell epitope repertoires within Dsg3 and Dsg1 suggests that similar peptides from both PV target antigens may be involved in disease progression and the evolution in autoreactive lymphocyte reactivity during the course of disease from one clinical subtype to another (mucosal PV to mucocutaneous PV).

## Results and Discussion

### Comparison of Dsg1 and Dsg3 Extracellular Domains

**Figure 1:**
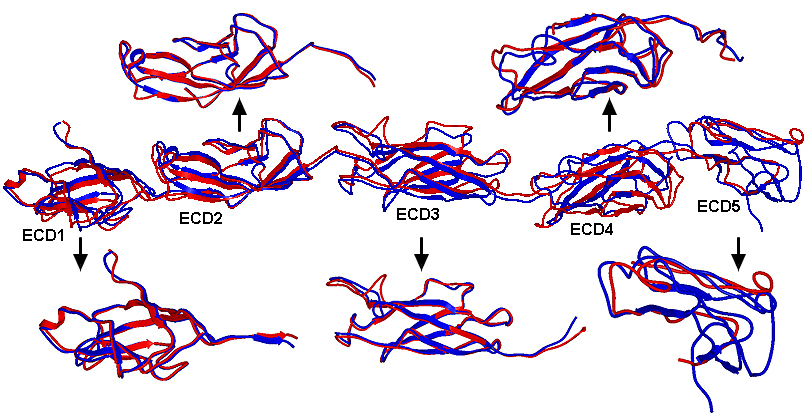
**Superposition of the structures of Dsg1 and Dsg3 extracellular domains**. Structural comparison of the Dsg1 (red) and Dsg3 (blue) extracellular domains. The r.m.s.d. of the entire ECD, ECD1, ECD2, ECD3, ECD4, and ECD5 are 5.56 Å, 1.03 Å, 1.09 Å, 1.94 Å, 3.76 Å, and 6.95 Å, respectively.

**Figure 2:**
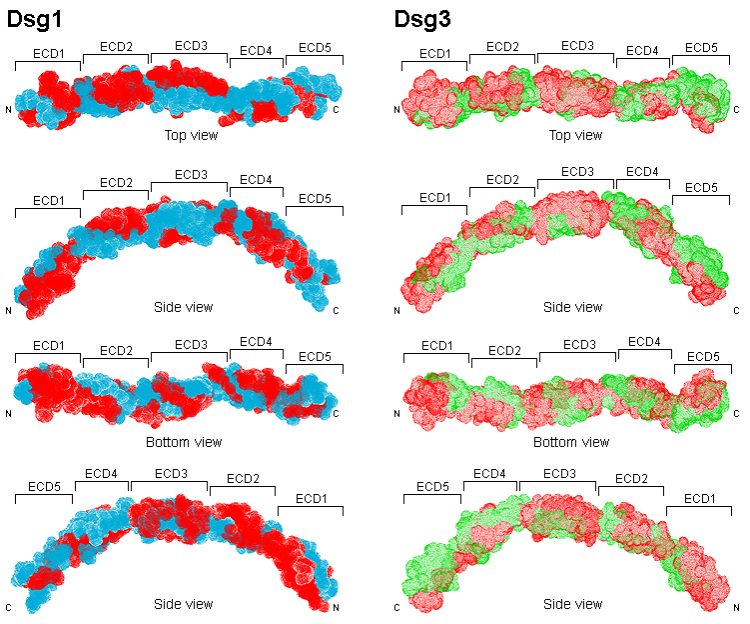
Molecular surface of Dsg1 and Dsg3 extracellular domains illustrating the positions of predicted DRB1*0402-specific immunological (T-cell epitope) hot spots. Locations of predicted immunological hot spots (colored red) for Dsg1 and Dsg3 extracellular domains are shown in surrounding views and numbered in accordance to their extracellular domains.

**Figure 3:**
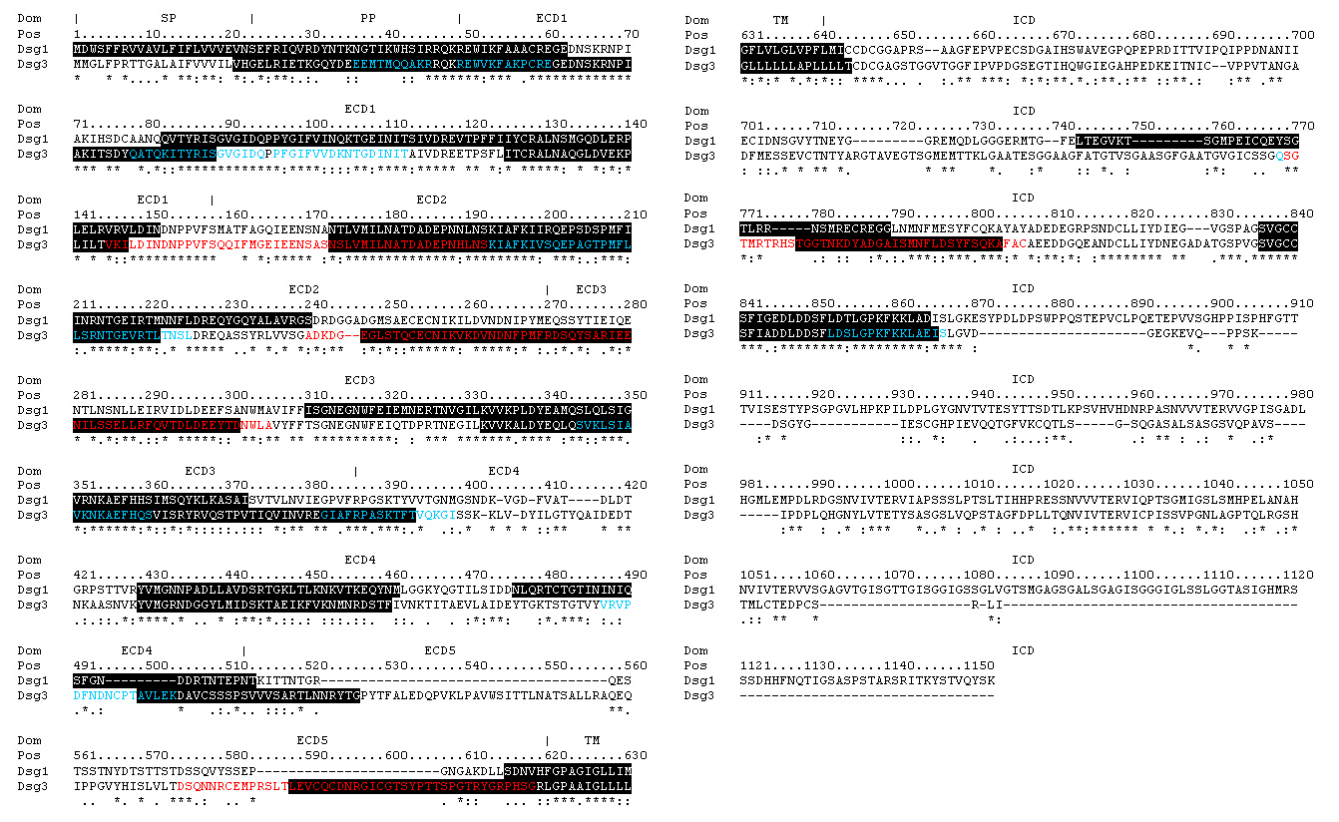
Predicted DRB1*0402-specific immunological hot spots for Dsg1 and Dsg3 glycoproteins. Multiple sequence alignment of Dsg1 and Dsg3. Predicted hot spots (threshold = -123 kJ/mol) shaded in black, experimental Dsg3 hot spots highlighted red, and experimentally confirmed DRB1*0402-specific Dsg3 peptides highlighted blue. Dsg1 – Signal Peptide, SP: 1–23; Propeptide, PP: 24–49; Extracellular domain 1, ECD1: 50–158; ECD2: 159–270; ECD3: 271–385; ECD4: 386–497; ECD5: 498–548; Transmembrane, TM: 549–569; Intracellular domain, ICD: 570–1049. Dsg3 – SP: 1–23; PP: 24–49; ECD1: 50–158; ECD2: 159–268; ECD3: 269–383; ECD4: 386–499; ECD5: 500–615; TM: 616–640; ICD: 641–999.

The Dsg3 extracellular domain (ECD) has an extensive surface area of 32133 Å ^2^. This surface area is ~3% larger than the Dsg1 ECD atomic accessible surface (31093 Å ^2^). The general folds of Dsg1 and Dsg3 ECDs are similar (Figures 1 and 2). In particular, the ECD1, ECD2 and ECD3 of Dsg1 and Dsg3 are well conserved with Cα r.m.s.d. of 1.03 Å, 1.09 Å and 1.94 Å, respectively. The main differences between Dsg1 and Dsg3 ECDs lie in ECD4 (Cα r.m.s.d. = 3.76 Å) and ECD5 (Cα r.m.s.d. = 6.95 Å). In ECD4, the most obvious difference between the structures involve the loop 411–420 of Dsg1 and loop 405–422 of Dsg3. The backbone of Gln410, Ala411 and Ile412 in Dsg1 are intercalated more deeply into the ECD4 interface than the corresponding residues in Dsg3. In ECD5, the loop 468–479 curled upward toward Dsg1, with differences of up to 2.18 Å between the corresponding positions in Dsg3 (loop 468–475). It appears that these regions may not contain high concentration of T-cell epitopes and do not contribute directly to the differences in T-cell specificities of the two PV target antigens (Figure 3). Nonetheless, these structural differences are solvent exposed and may interact with pemphigus autoAb.

### Dsg1 and Dsg3 Share Common Immunological Hotspots

The locations of Dsg1 and Dsg3 immunological hotspots are highly conserved across the ECD, TM and intracellular domain (ICD), despite limited sequence identity (54% identical, 72% similar). There are substantial overlaps between predicted and known immunological (T-cell epitope) hotspots (Dsg3 145–192, Dsg3 240–303, Dsg3 570–614) at the threshold -123 kJ/mol (Figure 2). At this threshold, the number of predicted Dsg3 hotspots are ten (residues 21–88, 125–147, 173–221, 245–299, 330–391, 435–456, 495–522, 584–640, 772–797, 830–859). Among these, one spans the signal peptide (SP), propeptide (PP) and the ECD; six are present in the ECD; one extends across the ECD, TM and ICD; while two hotspots are predicted to exist in the ICD (Figure 2). Similarly, nine hotspots were predicted for Dsg1 (residues 1–62, 82–151, 173–240, 310–372, 423–455, 470–498, 550–569, 656–688, 732–760), all of which overlap with those from Dsg3 (Figure 2). Collectively, these results suggest that the potential T-cell epitope repertoire encoded in Dsg1 and Dsg3 is substantially overlapping, and may help to explain the molecular basis underlying the observed inter-molecular spreading from Dsg3 to Dsg1 targets during the course of PV.

### Effects of Binding Registers in Peptide Selection

**Figure 4:**
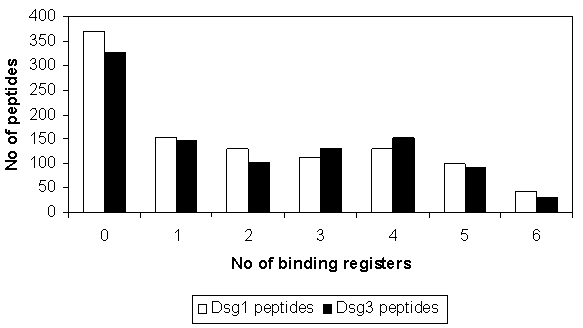
Number of predicted binding registers for Dsg1 and Dsg3 peptides bound to DRB1*0402. The frequency of Dsg1 (colored white) and Dsg3 (colored black) peptides docked to DRB1*0402 shown as a function of the number of predicted binding registers.

We investigated the effect of multiple contact regions or binding registers in Dsg3 peptides specific to DRB1*0402 [33]. Among 985 Dsg3 peptide sequences (including SP and PP derived sequences), 658 were predicted high-affinity binders with 77% containing two or more registers that can be docked into the binding groove of DRB1*0402. A similar number of high-affinity binders are predicted to exist within the Dsg1 glycoprotein, with analogous distribution of binding registers as illustrated in Figure 4. Of 1035 Dsg1 peptides, 665 were predicted high-affinity binders with 129 (~23%), 112 (~19%), 130 (~17%), 99 (~15%) and 43 (~6%) peptides possessing two, three, four, five and six registers, respectively.

### Redundancy Profiles of Predicted Binding Peptides

**Figure 5:**
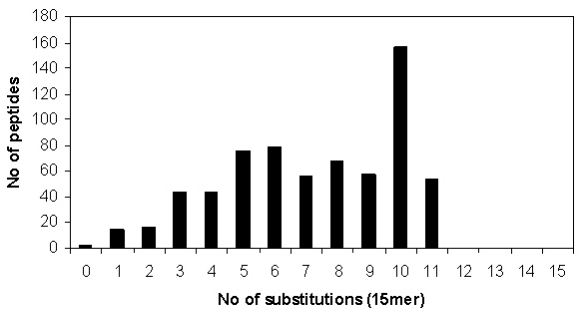
**Redundancy profile of predicted Dsg1 peptides to Dsg3 sequences**. The number of predicted high-affinity Dsg1 (15 mer) peptide sequences shown as a function of the number of substitutions from the highest identity peptide sequence derived from the Dsg3 proteome derived using a 15 mer sliding window.

The predicted Dsg1 high-affinity binding sequences were examined for their similarity with the Dsg3 proteome. Each Dsg1 (15 mer) sequence was used to probe the entire Dsg3 proteome for the highest identity Dsg3 (15 mer) peptide with the minimal number of substitutions. Figure 5 details the degree of conservation of Dsg1 predicted high-affinity binders with Dsg3 sequences. All predicted Dsg1 and Dsg3 (15 mer) binding peptides share at least four common residues along their primary sequences. It has been reported that PV and PF autoAbs can cross-react with Dsg1 and Dsg3 peptides with 75% identity [38]. Sequence alignment showed that 18% (or 122) of these peptides are highly conserved with at least 75% sequence identity. In this context, two peptides Dsg1_58–72 _CREGEDNSKRNPIAK and Dsg1_59–73 _REGEDNSKRNPIAKI near the N-terminus of ECD1 appear to be of particular interest, since they are fully conserved within the Dsg3 proteome and may represent the most likely antigenic link between self-directed responses to two distinct autoantigens (Dsg3 and Dsg1). A subset of patients with PV has been shown to have T cell reactivity to both Dsg3 and Dsg1 [39]. However, defined Dsg1 epitopes have not yet been determined.

## Conclusion

Although clinical evolution of disease expression is common in PV [3,4], our understanding of how T-cells are involved in the underlying autoreactive processes remains poor. Collectively, the results of this study provide interesting new insights into the possible mechanism of disease progression. Our data suggests that similar peptides from the two known PV autoantigenic targets may bind to DRB1*0402 and allow for intermolecular epitope spreading that lead to distinct morphological categories of PV – mucosal lesions only *vs*. mucocutaneous disease. Nonetheless, many other factors exist, such as IgG-activated intracellular signalling events [40], which may play a critical role in the complex disease mechanism and should also be explored. Recently, Lucchese and coworkers [38] discovered an immunodominant Dsg3 T-cell epitope Dsg3_49–60 _REWVKFAKPCRE that is highly reactive with both PV and PF sera, with 75% identity to Dsg1_49–60 _REWIKFAAACRE. In this context, our current approach of epitope mapping may prove useful in facilitating the systematic discovery of peptides that cross-react with both PV and PF autoreactive T-cells and serve as targets for potential therapeutic approaches that are efficient in both diseases. Further studies are necessary to determine the proportion of immunogenic epitopes that are capable of stimulating autoreactive lymphocytes from both pemphigus subtypes.

## Methods

The sequence of DRB1*0402 was obtained from IMGT-HLA database [41]. Dsg1 and Dsg3 sequence data were obtained from Swiss-Prot [42]. To identify suitable structural templates in the Protein Data Bank (PDB) [43] for model construction, a sequence similarity search was performed using BLASTP [44] and the highest quality templates (with the best resolution, highest sequence similarity and minimal number of missing residues) were selected among the returned hits. The crystal structure of DRB1*0401 (PDB code 1D5Z) was selected as template for DRB1*0402 (97.9% identity) [33], while the solution structures of C-cadherin (PDB code 1Q5C) and N-cadherin (PDB code 1NCJ) were used for constructing the extracellular domains of both Dsg1 and Dsg3.

### Model Building

The program MODELLER [45] was employed for comparative modeling of DRB1*0402, Dsg1 and Dsg3. Each model was constructed by optimally satisfying spatial constraints obtained from the alignment of the template structure with the target sequence and from the CHARMM-22 force field [46], and subsequently relaxed by conjugate gradient minimization, using the program Internal Coordinate Mechanics (ICM) [47,48].

### Peptide Docking

**Figure 6:**
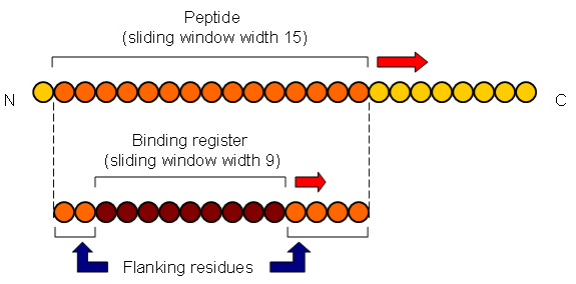
Generation of Dsg1 and Dsg3 peptide sequences for docking into DRB1*0402. Overlapping 15 mer peptides are generated from the Dsg1 and Dsg3 sequences. For each 15 mer peptide, an overlapping sliding window of size nine is applied to generate all combinations of binding registers to be modeled into the binding groove of DRB1*0402.

**Figure 7:**
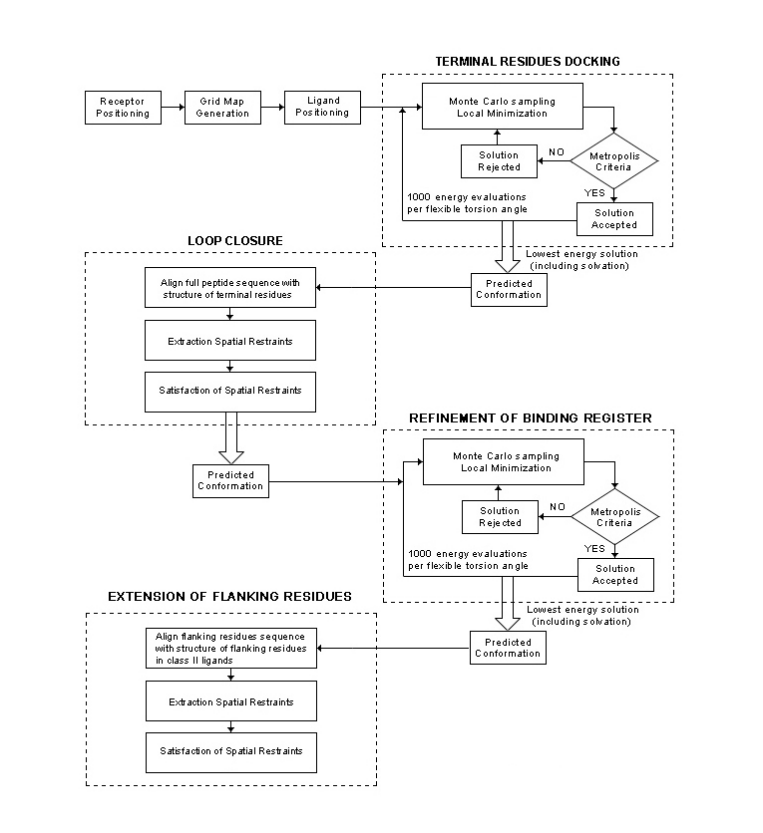
**Flowchart of the four-step docking procedure used in this work**. Peptides are docked into DRB1*0402 using a four-step procedure consisting of: (i) pseudo-Brownian rigid body docking of peptide fragments to the ends of the binding groove, (ii) central loop closure by satisfaction of spatial constraints, (iii) refinement of the backbone and side-chain atoms of the core recognition residues and receptor contact regions and (iv) extension of flanking peptide residues by satisfaction of spatial constraints.

Overlapping 15 mer peptides are generated from the Dsg1 and Dsg3 sequences. An overlapping sliding window of size nine is applied to each 15 mer peptide to generate all combinations of binding registers to be modeled into the binding groove of DRB1*0402 (Figure 6). A total of 1035 Dsg1 15 mer peptides (6210 binding registers) and 985 Dsg3 15 mer peptides (5910 binding registers) were generated and used in the current analysis. Docking was performed using a standard protocol as previously described [33-35], consisting of (i) pseudo-Brownian rigid body docking of peptide fragments to the ends of the binding groove, (ii) central loop closure by satisfaction of spatial constraints, (iii) refinement of the backbone and side-chain atoms of the core recognition residues and receptor contact regions within 4.00 Å radius, and (iv) extension of flanking peptide residues by satisfaction of spatial constraints (Figure 7).

### Empirical Free Energy Functions

The scoring function presented in this study is based on the free energy potential in ICM [47,48]. Computation of the binding free energy was performed according to previous similar works [36,37] based on the difference between the energy of the solvated complex and the sum of the energy of the solvated receptor and that of the peptide ligand, followed by optimization using experimental IC_50 _values. The DRB1*0402 model optimization, training and testing were described earlier [33].

### Immunological Hotspot Prediction

In the present study, 'immunological hotspots' are defined as antigenic regions of up to 30 amino acids based on the sum of predicted binding energies of the top four binders within a window of 30 amino acids [49,50]. Where available, predicted hotspots were validated using available experimentally determined sites (Figure 3).

### Accessible Surface Areas

Solvent accessible surface areas were calculated with the program NACCESS [51] by tracing out the maximum permitted van der Waals' contact that is covered by the center of a water molecule (1.40 Å probe radius) as it rolls over the surface of the protein.

## Authors' contributions

JCT carried out the computational simulation studies. JCT and AAS participated in the experimental design, data interpretation and drafted the manuscript.
